# Psychosocial emergency care in times of COVID-19: the Essen University Hospital concept for corona-infected patients, their relatives, and medical staff

**DOI:** 10.1007/s00420-020-01580-z

**Published:** 2020-09-22

**Authors:** Vanessa Rentrop, Johanna Sophie Schneider, Alexander Bäuerle, Florian Junne, Nora Dörrie, Eva-Maria Skoda, Manfred Schedlowski, Bernhard Mallmann, Anke-Verena Benecke, Hannah Kohler, Monja Gerigk, Per Teigelack, Tobias Emler, Norbert Scherbaum, Gertraud Gradl-Dietsch, Karin Scheer, Martin Teufel

**Affiliations:** 1 Department of Psychosomatic Medicine and Psychotherapy, LVR University Hospital Essen, University of Duisburg-Essen, 45147 Essen, Germany; 2 Department of Psychosomatic Medicine and Psychotherapy, University Hospital Tübingen, Eberhard Karls University Tübingen, 72076 Tübingen, Germany; 3 Institute of Medical Psychology and Behavioral Immunobiology, University Hospital Essen, 45147 Essen, Germany; 4 Department of Clinical Neuroscience, Osher Center for Integrative Medicine, Karolinska Institutet, 171 77 Stockholm, Sweden; 5 Palliative Medicine, University Hospital Essen, 45147 Essen, Germany; 6 Institute of Patient Experience, University Hospital Essen, 45147 Essen, Germany; 7 Strategic Management, University Hospital Essen, 45147 Essen, Germany; 8 Department of Psychiatry and Psychotherapy, LVR University Hospital Essen, Medical Faculty, University of Duisburg-Essen, 45147 Essen, Germany; 9 Department of Child and Adolescent Psychiatry, Psychotherapy and Psychosomatics, LVR University Hospital, University of Duisburg-Essen, 45157 Essen, Germany; 10 Hospice Care, University Hospital Essen, 45157 Essen, Germany

**Keywords:** Coronavirus, Psychosocial emergency care, Psychological support strategies, Medical staff

## Abstract

Due to the SARS CoV-2-virus (COVID-19), anxiety, distress, and insecurity occur more frequently. In particular, infected individuals, their relatives, and medical staff face an increased risk of high psychological distress as a result of the ongoing pandemic. Thus, structured psychosocial emergency concepts are needed. The University hospital of Essen has taken up this challenge by creating the PEC concept to reduce psychosocial long-term consequences for infected patients, relatives, and medical staff at the university hospital. The concept includes professional medical as well as psychological support to convey constructive coping strategies and the provision of adequate tools such as the low-threshold online training program (CoPE It), which is accessible via the webpage www.cope-corona.de.

## To the Editor

**Fig. 1:**
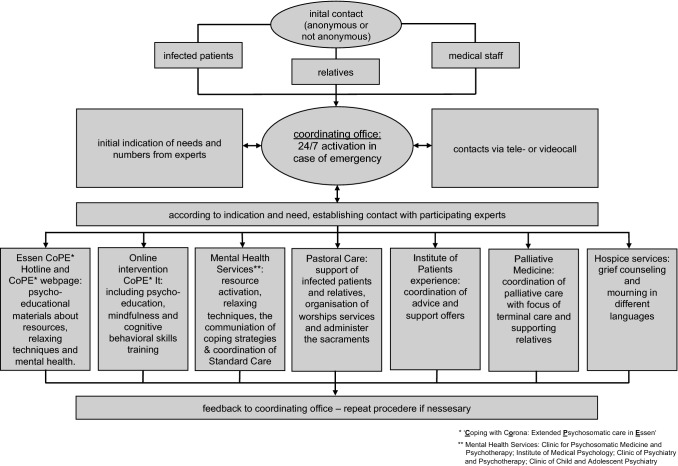
Psychosocial emergency concept in Essen: components and need-based paths

Since the beginning of the year, the SARS CoV-2 virus (COVID-19) had a significant impact on the lives of the majority of people all around the world (Zhu et al. 2020; Tedros 2020). As reported by the Robert Koch Institute, the central health institution of the federal German government, as of 01 September 2020, there are currently 243,599 confirmed cases of COVID-19 infections in Germany, of which 59,023 were diagnosed in the federal state of North Rhine-Westphalia (NRW) (Robert Koch Institute 2020). The protective restrictions taken by the government (e.g. physical distancing, private quarantine) and immediate effects of the virus itself, increase the risk of psychological distress for the general population but in particular for specific risk groups (Brooks et al. 2020). In addition to the needs of the infected patients themselves, their relatives in particular are confronted with worries and concerns about the state of health and the medical condition of the affected individual and also need help and support for example due to visiting bans in hospitals. Moreover, the pandemic has significantly changed the working conditions of medical staff by causing the following problems: longer working hours, limited protective equipment, contact with infected persons, and, therefore, an increased risk of contracting COVID-19. This is highlighted by the rising numbers of deaths among medical staff worldwide. Furthermore, due to the limited capacity within intensive care, it seems relevant that the medical staff is prepared to decide which patients will receive further treatment. These decisions will lead to ethical concerns, challenging the medical staff. The critical condition of many patients, the suffering of relatives—also in the context of massive numbers of terminally ill patients—and the still insufficient scientific knowledge regarding the treatment of infected patients could push medical staff to their limits and enhance the occurrence of psychological symptoms (Huang et al. 2020). To maintain the work ability of healthcare professionals during the crisis is essential. Previous studies show that the possibility to use telemedicine during the COVID-19 pandemic is a precious resource to help people without physical contact (Chou et al. 2020). To support the above-mentioned risk groups, a psychosocial emergency care (PEC) concept has been developed in Essen, Germany. Other concepts focus primarily on supporting psychologically burdened people within the community. However, the PEC focuses mainly on infected patients, their relatives, and medical staff. Previous guidelines and scientific texts deal with the establishment of a psychosocial emergency care concept for prevention and early recognition of the psychosocial consequences of stressful events, the provision of appropriate support for processing experiences and, if necessary, the treatment of mental stress (Deffner et al. 2020). The entire procedure of the PEC is shown in Fig. 1.

As a first step, infected patients, relatives, and medical staff of the Essen University Hospital can contact the coordinating office by telephone either anonymously or not anonymously if required. After primary assessment and the allocation trough the coordinating office, the person who called is subsequently contacted by an expert, a psychologist or physician specialized in psychosomatic medicine. Appointments are held via telephone or video calls. These initial assessments aim to capture the extent and scope of the issue leading to the indicated need. The required number of professionals is then activated. For patients and relatives, the intervention consists predominantly of psychotherapeutic care to achieve stability through psycho-education, mindfulness, and cognitive behavioral skills. It also aims at providing help with topics such as stabilizing interventions, resource activation, relaxation techniques, and coping strategies. For medical staff, recommendations are given to deal with periods of high mental stress, including optional external moderation for highly stressed teams and managers.

Additionally, self- and third-party risks are examined at the current time and in case of an emergency, first aid can be activated. If necessary, further treatment in regular psychological care is discussed. Each call is documented pseudonymously to record the content of the telephone calls. Subsequently, feedback is given to the coordinating office, to allow adaptions of the offers to the needs of the individuals seeking help. The coordinating office has the opportunity to involve additional resources. As an additional strategy for low-threshold crisis intervention, there is also the possibility of using a new online intervention, which was developed to reduce psychological distress and worries and improve the feeling of control during the pandemic by supporting burdened individuals in a self-help manner named CoPE It (Bäuerle et al. 2020a, b). To reach and support as many affected people as possible, CoPE It is offered anonymously and without costs. The psychological burden of COVID-19 for infected people, relatives, and medical staff is evident and can be seen throughout the current literature. Structured low-threshold psychosocial emergency care needs to be established in due time. By implementing such a concept in the University hospital Essen, those needs can be met and further adverse developments concerning COVID-19 can be countered.
